# An emerging multi-omic understanding of the genetics of opioid addiction

**DOI:** 10.1172/JCI172886

**Published:** 2024-10-15

**Authors:** Eric O. Johnson, Heidi S. Fisher, Kyle A. Sullivan, Olivia Corradin, Sandra Sanchez-Roige, Nathan C. Gaddis, Yasmine N. Sami, Alice Townsend, Erica Teixeira Prates, Mirko Pavicic, Peter Kruse, Elissa J. Chesler, Abraham A. Palmer, Vanessa Troiani, Jason A. Bubier, Daniel A. Jacobson, Brion S. Maher

**Affiliations:** 1 GenOmics and Translational Research Center and; 2 Fellow Program, RTI International, Research Triangle Park, North Carolina, USA.; 3 The Jackson Laboratory, Bar Harbor, Maine, USA.; 4 Biosciences Division, Oak Ridge National Laboratory, Oak Ridge, Tennessee, USA.; 5 Whitehead Institute for Biomedical Research, Massachusetts Institute of Technology, Cambridge, Massachusetts, USA.; 6 Department of Psychiatry, UCSD, La Jolla, California, USA.; 7 Division of Genetic Medicine, Vanderbilt University Medical Center, Nashville, Tennessee, USA.; 8 Institute for Genomic Medicine, UCSD, La Jolla, CA, USA.; 9 Geisinger College of Health Sciences, Scranton, Pennsylvania, USA.; 10 Department of Mental Health, Johns Hopkins Bloomberg School of Public Health, Baltimore, Maryland, USA.

## Abstract

Opioid misuse, addiction, and associated overdose deaths remain global public health crises. Despite the tremendous need for pharmacological treatments, current options are limited in number, use, and effectiveness. Fundamental leaps forward in our understanding of the biology driving opioid addiction are needed to guide development of more effective medication-assisted therapies. This Review focuses on the omics-identified biological features associated with opioid addiction. Recent GWAS have begun to identify robust genetic associations, including variants in *OPRM1*, *FURIN*, and the gene cluster *SCAI*/*PPP6C*/*RABEPK*. An increasing number of omics studies of postmortem human brain tissue examining biological features (e.g., histone modification and gene expression) across different brain regions have identified broad gene dysregulation associated with overdose death among opioid misusers. Drawn together by meta-analysis and multi-omic systems biology, and informed by model organism studies, key biological pathways enriched for opioid addiction–associated genes are emerging, which include specific receptors (e.g., GABAB receptors, GPCR, and Trk) linked to signaling pathways (e.g., Trk, ERK/MAPK, orexin) that are associated with synaptic plasticity and neuronal signaling. Studies leveraging the agnostic discovery power of omics and placing it within the context of functional neurobiology will propel us toward much-needed, field-changing breakthroughs, including identification of actionable targets for drug development to treat this devastating brain disease.

## Introduction

Opioid misuse, addiction, and associated overdose deaths remain major public health crises. Worldwide, more than 60 million people currently misuse opioids (1), 21 million people have opioid use disorder (OUD) (2), and 125,000 people died of an opioid overdose in 2019 (3). In the United States, more than 9 million people reported misusing opioids in 2020, 6–7 million currently have OUD (4), and 109,000 opioid-related overdose deaths occurred in 2022 (5). These figures have steadily risen for more than a decade.

Despite the urgent need to curb the opioid epidemic, pharmacological treatment options are limited in their number, use, and effectiveness. Currently, methadone, buprenorphine, and naltrexone are the only FDA-approved medications for treating opioid addiction, and fewer than 1 in 5 individuals with OUD receive such medication-assisted treatment (MAT) (6). MAT is efficacious in reducing opioid use and overdose deaths while in treatment and promoting retention in treatment relative to no-MAT (7). However, a large proportion of patients with OUD on MAT relapse, with fewer than half remaining in treatment at 1 to 3 months of follow-up (8, 9). To reduce relapse in patients with OUD, we must make a dramatic leap forward in our understanding of the biology that drives opioid addiction to guide the development of more effective MAT.

This Review focuses on the omics-identified biological features associated with opioid addiction. “Opioid addiction” is used in this Review as a general term encompassing a variety of highly correlated phenotypes (genetic correlation >0.9), including misuse of prescription opioids, frequent use of illicit opioids, OUD, and qualifying for methadone maintenance (10). We will use this term to encompass the variety of phenotypes used across omics studies, excluding opioid overdose death. We use a more specific term herein when the cited research focused on a narrow phenotype; for example, we use opioid misuse to mean use of prescription opioids for purposes other than prescribed, use of someone else’s prescription, or to get high; we use OUD in reference to Diagnostic and Statistical Manual (DSM) or ICD–electronic health record diagnosis of a substance use disorder for opioids; and we use opioid overdose death to indicate a death that is a attributed to an overdose from any opioid or combination of them. We define omics as a range of technologies and analytic approaches that characterize biological features across domains (e.g., genetic variants, genes, RNAs, histone modifications, methylation, proteins) that are evaluated with hypothesis-free analytic approaches (e.g., genome- or transcriptome-wide statistical tests for association with a phenotype). Most often these studies are conducted using a single omic data type (e.g., genome-wide genotypes) in humans. However, there is increasing interest in, and opportunities for, cross-species and multi-omic studies, which also inform this Review.

Over the past 4 years, GWAS of opioid addiction have begun to reach sufficient size to identify robust genetic associations that are consistent across studies and related phenotypes. In parallel, there is an increasing number of omics studies of postmortem human brain tissue, examining a variety of biological features across different brain regions. These studies have begun to identify broad gene dysregulation driven by both genetics and exposures associated with opioid-overdose death among people who misuse opioids. Drawn together by meta-analysis and multi-omic systems biology in humans, and informed by model organism studies, this Review summarizes emerging key biological pathways for opioid addiction.

## GWAS

Beginning in the mid-2000s, the field of genetics generally and the addiction subfield moved away from family-based linkage studies in favor of case/control GWAS enabled by the development of low-cost high-throughput genotyping technology. Since then, genetics has been transformed from a field with only a handful of robustly identified associations between genetic variants and phenotypes to one where tens of thousands of variants have been associated with more than a thousand phenotypes (11). The field may have even seen the first case of a fully saturated characterization of the genetic contributions to a phenotype with a recently published study of height (12). While some early successful studies, such as those of irritable bowel syndrome, gave rise to hopes for generally rapid identification of key genetic drivers of disease, it became evident that, for many phenotypes, including complex behavioral disorders like addiction, there is a discovery “dead zone” where little is found until sufficient sample size is reached, and growth in the number of variant associations becomes a steady feature of ever-larger GWAS (13). In the field of addiction, the growth in identified variant associations began with studies of nicotine dependence (14) and is most prominent for cigarette smoking and alcohol misuse phenotypes (15). The most recent GWAS and Sequencing Consortium of Alcohol and Nicotine use iteration includes sample sizes up to 3.3 million and identifies over 1,300 loci associated with ever smoking cigarettes (15).

For opioid addiction, 38%–61% of the population variability is attributable to genetic factors in twin-based studies and 11%–18% from SNP-based heritability (10, 16, 17). However, it is only in the past few years that opioid addiction cases in GWAS have exceeded 10,000. With the growth in sample size, we have begun to see robust associations identified across studies and definitions of cases and controls in opioid addiction GWAS.

As of 2023, 10 GWAS of opioid addiction reported genome-wide significant associations at the variant- or gene-level of analysis (Table 1). The largest number of independent loci, 14, were identified by Kember et al. (17), who conducted the largest cross-ancestry meta-analysis, including European, African, and Hispanic ancestries. Across GWAS, the most consistently identified locus resides in the mu opioid receptor gene (*OPRM1*) and is commonly tagged by SNP rs1799971 or a variant in strong linkage disequilibrium (LD) with it, such as rs9458500. Indeed, because of the extensive haplotype that includes these *OPRM1* variants, it is unclear which variant is causal and what mechanism underlies this signal (10). Additional loci/genes that have been identified in multiple GWAS include furin paired basic amino acid cleaving enzyme (*FURIN*) and a cluster of genes on chromosome 9, across which significant variants in LD reside, including suppressor of cancer cell invasion (*SCAI*), phosphatase 6 catalytic subunit gene (*PPP6C*), and Rab9 effector protein with Kelch motifs (*RABEPK*). One caveat to this consistency of associations between opioid addiction and these loci/genes is that the studies reporting them include overlapping data (e.g., from the Million Veteran Program); i.e., they are not independent replications. In terms of independent replication, Gaddis et al. (10) found nominal support in their GWAS of opioid addiction for two signals reported by Sanchez-Roige et al., who examined problematic prescription opioid use: rs640561 (*P* = 0.009) and *PTPRF* (*P* = 0.026) (18). Although these results are important steps forward, it is important to note the limited diversity of participants included in these GWAS. A large majority of cases were of European ancestry (~75%), with 22% of African, 1.7% of Latino/Hispanic, and 0.3% of Asian ancestry (Table 1). Additionally, no GWAS reported sex-stratified analyses, though differences in risks for opioid addiction by sex are known (19, 20).

To assess the degree to which genetic associations are shared across phenotypes, recent GWAS conducted a number of follow-up analyses, including LD score regression (21), phenome-wide association studies (17), and genomic structural equation modeling (22). Similar to a broader analysis of substance use disorders (23), these analyses of opioid addiction (10, 16, 17) showed high degrees of genetic correlation between opioid addiction and other drug addiction phenotypes and, to a lesser degree, genetic correlations with psychiatric disorders (e.g., bipolar) and other medical conditions (e.g., type 1 diabetes and skin cancer) (17).

## Omics of gene regulation in the human brain

Complementing GWAS discoveries of variants affecting risk of opioid addiction, studies of gene regulation can identify evidence of genetic and environmental influences that contribute to disease. By assaying differences in gene regulation such as DNA methylation (DNAm), chromatin modification, gene expression, and alternative mRNA splicing in disease-relevant tissues, these studies reveal critical insights into functional mechanisms contributing to opioid addiction.

**Figure 1:**
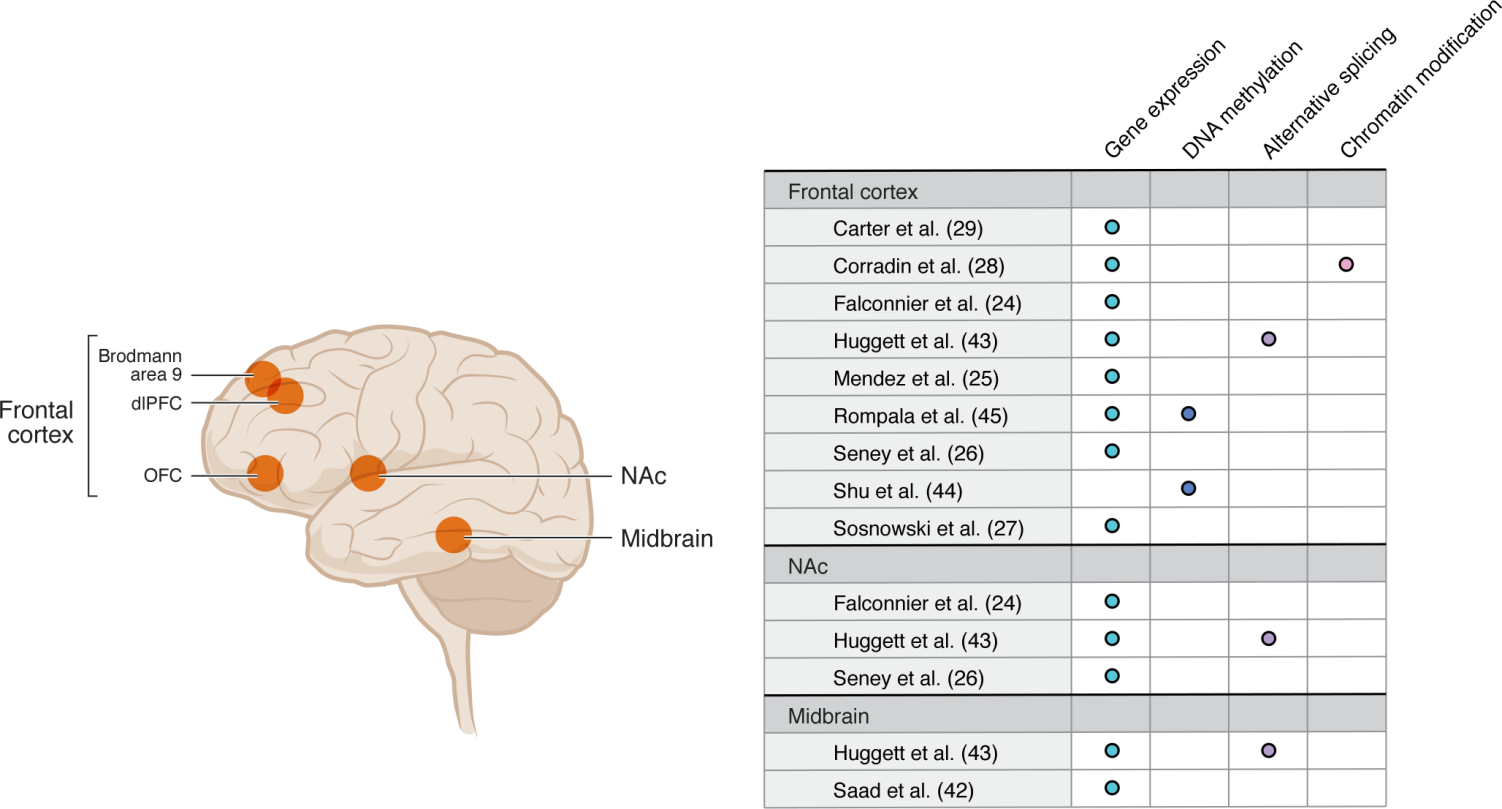
Studies of gene regulation associated with opioid addiction phenotypes in postmortem human brain. OFC, orbitofrontal cortex.

Falconnier et al. (24) recently published the first systematic Review of functional genomics across model organisms and human tissue studies of opioid addiction–related phenotypes. Identifying 73 studies that met their criteria (62 model organism and 11 human), they observed substantial heterogeneity in study design with respect to the phenotypes, brain regions, and omics technologies that were used. These authors found low consistency of results across studies, likely due to the heterogeneity and consistently modest-to-small sample sizes. In one of their most rigorous evaluations, they focused on results from 24 studies of differential gene expression in five of the most studied brain regions. Here, they identified 11 genes that were present in at least three studies as differentially expressed, with the same direction of effect: *Cdkn1a, Slc2a1, Fkbp5, Sult1a1, Arrdc3, Ccdc117, Plin4, Wscd1, Arid5b, Pla2g3*, and *Tsc22d3*. In this section of our Review, we focus on a subset of recent human studies that used sequencing technology, including some studies not captured in Falconnier et al. (24) (Figure 1). Of note, studies of human brain reviewed by Falconnier et al. and those reviewed below used bulk tissue technology to investigate gene dysregulation associated with opioid addiction.

Several studies have performed RNA-Seq on human postmortem brain tissue from individuals with OUD or opioid overdose with evidence of opioid misuse, which they compared with brains from individuals acting as controls to discover differentially expressed genes (DEGs) that may be involved in responses to acute or chronic opioid use. Mendez et al. (25) performed RNA-Seq and proteomic analyses in tissue from Brodmann area 9 in 29 individuals with OUD in the case group and 18 individuals acting as controls. These authors identified nearly 400 DEGs and over 200 differentially expressed proteins and reported that four genes (*LGALS3, SLC2A1, PCLD1,* and *VAMP1*) were differentially expressed in RNA and protein. Seney et al. (26) found 567 and 1,305 DEGs in dorsolateral prefrontal cortex (dlPFC) and nucleus accumbens (NAc) tissues, respectively, from 20 individuals with OUD and 20 individuals acting as controls. Downstream, concordance and pathway analyses provided evidence for multiple inflammatory processes. Sosnowski et al. (27) compared gene expression in a dlPFC sample from 72 individuals with opioid intoxication as a cause of death and 81 individuals acting as controls, which showed significant underexpression of *NPAS4* in samples from the case group. Pathway analyses in this study did not provide compelling evidence for any gene set. As part of a study of both differential H3K27ac acetylation and gene expression in dlPFC of opioid misusers who had overdosed and individuals acting as controls, Corradin et al. (28) identified 10 DEGs in a sample of 24 opioid overdose cases among people who misuse opioids and 27 individuals acting as controls, including *ARC, ERG2, DUSP4,* and *DUSP6*. The most statistically significant gene set enrichment was found for the MAP kinase pathway in the Gene Ontology molecular function annotation.

Leveraging the publication of four studies of differential gene expression in dlPFC assessed by RNA-Seq for similar opioid overdose phenotypes (25–28), Carter et al. (29) conducted a meta-analysis of a combined sample of 283 (cases = 170, controls = 113). They found 335 DEGs, 66 of which overlapped with those genes previously reported, including *DUSP2, DUSP4, DUSP6, EGR1, EGR4, ARC,* and *NPAS4*. Enrichment analyses of the set of 335 DEGs using several ontologies identified significant enrichment across an interconnected set of receptors and signal transduction pathways that include the orexin and receptor tyrosine kinase (RTK) to ERK/MAPK pathways linking to effectors of neuronal plasticity. It is noteworthy that the orexin pathway is the target of ongoing pharmacotherapy studies for opioid addiction (30). Orexin may play a role in OUD, with substantial evidence from preclinical models of its role in key processes in the addiction process, across multiple brain regions, largely via study of the orexin-1 receptor (OxR1). Experimental manipulation of orexin signaling impacts withdrawal from morphine (31), OxR1 antagonists reduce motivation for fentanyl and oxycodone (32), and orexin-KO mice exhibit reduced risk for morphine dependence (33). The likely mechanism of action is through orexin’s effect on the cAMP response element-binding protein (CREB) intracellular signaling pathway (34). In humans, an orexin receptor antagonist limits withdrawal, craving, and sleep disturbances during medication-assisted withdrawal (35). When evaluating 10 of the 11 genes reported by Falconnier et al. (24) in the results from the Carter et al. meta-analysis (29), only *ARRDC3* passed a Bonferroni-corrected *P* value threshold (*P* = 0.00035), while *WSCD1* was nominally significant (*P* = 0.032); one of the Falconnier et al. genes had been filtered out during quality control processing in Carter et al.

Alternative splicing of genes previously linked to addiction, such as *DRD2* and *OPRM1,* has been linked with multiple substance use disorders (36–41). Data from Seney et al. (26) and Saad et al. (42) were used by Huggett et al. (43) to evaluate the role of alternative splicing in OUD. This analysis identified 788 differentially spliced genes, including 8 in the Kyoto Encyclopedia of Genes and Genomes (KEGG) morphine addiction pathway (https://www.kegg.jp/). Most genes differentially expressed in individuals with OUD versus individuals acting as controls (*n* = 922) were not differentially spliced. That 30 genes were identified through both differential expression and splicing analyses suggests that differential splicing analysis has the potential to reveal novel genes linked to OUD.

DNAm studies of OUD have been done in the blood, orbital frontal cortex, and prefrontal cortex (44–46). A study of 140 women with opioid dependence and 80 opioid-exposed control samples identified three CpG sites with genome-wide significant differences in methylation (46). The most notable finding was cg21381136 in *RERE*, a gene harboring variants associated with several psychiatric/behavioral traits and which is involved in cell survival, chromatin remodeling, and transcriptional regulation during development. Shu et al. (44), in the same sample as that used by Sosnowski et al. (27), examined array-based differential methylation between individuals after opioid overdose death and individuals with other causes of death. The authors reported that no single site reached genome-wide significance, although a set of 13 genes was reported as “interesting,” including Netrin-1, a gene involved in κ opioid receptor activity. The authors also reported an association between advanced epigenetic age and acute opioid intoxication at death.

In addition to DNAm (5mC), DNA hydroxymethylation (5hmC), which is strongly enriched in neurons, has also been evaluated in the context of OUD. Here, neurons were isolated from the orbital frontal cortex of 12 OUD and 26 control samples (45). A total of 397 differentially methylated CpG sites were identified for 5mC and 1,740 for 5hmC. Genes linked with 5hmC alterations were significantly enriched for putative gene targets identified by Deak et al. in an OUD GWAS (16). Finally, Corradin et al. (28) conducted an analysis of chromatin modification using H3K27ac to identify alterations in gene regulation in individuals after opioid overdose death and individuals with other causes of death using neuronal nuclei isolated from 102 human postmortem dlPFC tissue samples. This study identified 388 genome-wide changes in acetylation, including hypoacetylation at the promoter of *DUSP4*. It also identified individual-specific hypoacetylation events that varied across opioid overdose cases. Using 3-dimensional chromatin interaction datasets, these individual-specific hypoacetylation events were found to frequently interact with the same target promoter, suggesting a convergence of epigenetic variation for a subset of gene targets. These investigators identified five common target genes with the most substantial convergence of hypoacetylation events across samples: *GABBR2, ASTN2, ENOX1, DUSP4,* and *KCNMA1*. Genes with the most convergence of hypoacetylation events were frequently found proximal to the top SNPs associated with OUD.

## Multi-omics for systems biology

To date, overlap in genetic variants and gene dysregulation that are associated with opioid addiction has appeared limited and has not been comprehensively evaluated. However, an increasing number of recent studies collectively showcase the power of multi-omic integration strategies in uncovering the molecular mechanisms underlying OUD and related conditions. Initial steps in this direction are exemplified by the studies characterized in Table 2. Each study employed a unique combination of data types and integration methods to explore different facets of opioid addiction, from genetic predispositions to epigenetic and transcriptomic changes. Several GWAS (10, 16–18) used biological annotation tools, such as FUMA (47), MAGMA (48), and H-MAGMA (49) as well as predicted gene expression (S-PrediXcan, ref. 50), to explore the functional consequences of the identified SNPs, linking variants to specific genes and pathways that suggest biological processes underlying OUD (e.g., colocalization analyses and cell-type enrichment analyses linking GWAS findings to SUD relevant areas of the brain). Seney et al. (26) integrated RNA-Seq data from the dlPFC and NAc with GWAS data and cell-type deconvolution to explore transcriptional changes in OUD. The study utilized differential expression analysis and weighted gene coexpression network analysis (WGCNA) to identify OUD-specific transcriptional networks. Integrating these data with GWAS revealed genetic liabilities associated with psychiatric phenotypes linked to OUD. Additionally, cell-type deconvolution provided insights into the role of microglia in opioid-induced neuroplasticity. This approach highlighted the involvement of neuroinflammation and synaptic remodeling in OUD pathophysiology. Other studies, like those by Mendez et al. (25) and Liu et al. (51), focused on integrating RNA-Seq data with DNAm and/or proteomic data to understand how genetic and epigenetic changes contribute to OUD. These integrative analyses uncovered critical insights into the complex molecular landscape of OUD, revealing that astrocyte and glial cell dysregulation, alongside alterations in synaptic vesicle formation, are key contributors to the disorder’s pathology. Corradin et al. (28) employed a distinctive integration strategy by combining ChIP sequencing (ChIP-Seq) data, which focused on H3K27 acetylation, with RNA-Seq and promoter-capture Hi-C data. This approach allowed the researchers to map long-range chromatin interactions and link distal regulatory elements to their target genes. By correlating changes in enhancer activity (identified through ChIP-Seq) with gene expression changes (from RNA-Seq), the study provided insights into the regulatory networks disrupted in opioid overdose. The integration of chromatin interaction data further refined the understanding of how epigenetic changes at enhancers translate into gene expression alterations.

Newer tools for integrating and analyzing multi-omic data hold promise to significantly advance the field, including Similarity Network Fusion (SNF) (52), Joint and Individual Variation Explained (JIVE) (53), Multi-Omics Factor Analysis (MOFA) (54), Gene set Refinement through Interacting Networks (GRIN) (55, 56), and Multiplex Embedding of Networks for Team-Based Omics Research (MENTOR) (57, 58), each offering distinct strengths and applications. SNF constructs and fuses similarity networks from multiple omic data types to uncover shared patterns and clusters, making it particularly effective for identifying disease subtypes in studies involving diverse biological datasets. In contrast, JIVE decomposes multi-omic data into shared components, capturing variation common across all data types and individual components, highlighting unique variation specific to each omic layer. This approach allows researchers to discern both common and unique patterns, providing insights into shared biological mechanisms and specific differences across datasets. MOFA identifies latent factors that explain variation across different omics datasets, using a probabilistic framework to model the joint structure of the data. This makes MOFA particularly flexible for integrative analyses in systems biology and personalized medicine, where uncovering molecular signatures and disease subtypes is essential. SNF, JIVE, and MOFA are designed to use datasets where the different omics layers are all generated from the same individuals and are not focused on mechanistic interpretation. While GRIN and MENTOR also support integration of omics data layers from the same cohort, importantly, they also support the integration of different omics layers collected from different cohorts and are focused on mechanistic interpretation, all of which greatly expands their utility.

GRIN and MENTOR further stand out for their use of Random Walk with Restart (RWR) and multiplex networks built from large amounts of publicly available data, which therefore represent millions of lines of evidence about gene/protein interaction and regulation, providing a powerful combination for refining and interpreting complex gene sets. GRIN is designed to filter large gene sets derived from GWAS and omics datasets by leveraging the mechanistic connections within multiplex networks. GRIN first applies RWR to rank genes based on their connectivity within the network, effectively filtering out potential false positives and retaining those genes that are mechanistically connected. MENTOR takes over to identify functionally and mechanistically connected subsets of genes within the refined set. By integrating multiple layers of biological evidence through multiplex networks, MENTOR enables the discovery of functionally related gene clusters as clades in the overarching structure of a dendrogram that serves as an elegant abstraction of the complexity encoded in the multiplex network topology. To the best of our knowledge, GRIN and MENTOR are the only ones of these methods to have been utilized on omic datasets associated with substance use disorders (56, 58).

An application of this approach to opioid addiction is found in Sullivan et al. (56), where RWR analysis yielded a dense network of 211 genes, which implicated the dysregulation of ERK/MAPK signaling, and Akt, BDNF, and other pathways. A resulting conceptual model of 45 genes from this larger network links these pathways with genes affecting cell receptor function identified in GWAS (e.g., *OPRM1* and *FURIN*) and orexin and tyrosine kinase receptors through MEK/ERK/MAPK signaling to affect neuronal plasticity that was highlighted in the differential gene expression meta-analysis of opioid overdose deaths in human dlPFC described by Carter et al. (29).

## Model organism omics

As reviewed above, recent GWAS, omic studies of gene regulation, and multi-omic systems biology studies in human populations have advanced our understanding of the genetics and putative mechanisms of OUD by identifying variants and genes statistically associated with opioid addiction phenotypes (10, 16–18, 29, 56, 59). However, to uncover the biological relevance of these regions of the genome, a deeper understanding of the underlying molecular mechanisms and neurological pathways is required. Model organisms are important tools to help bridge the gap between the identification of putative genetic targets in humans and understanding how they work (reviewed in refs. 24, 60).

**Figure 2:**
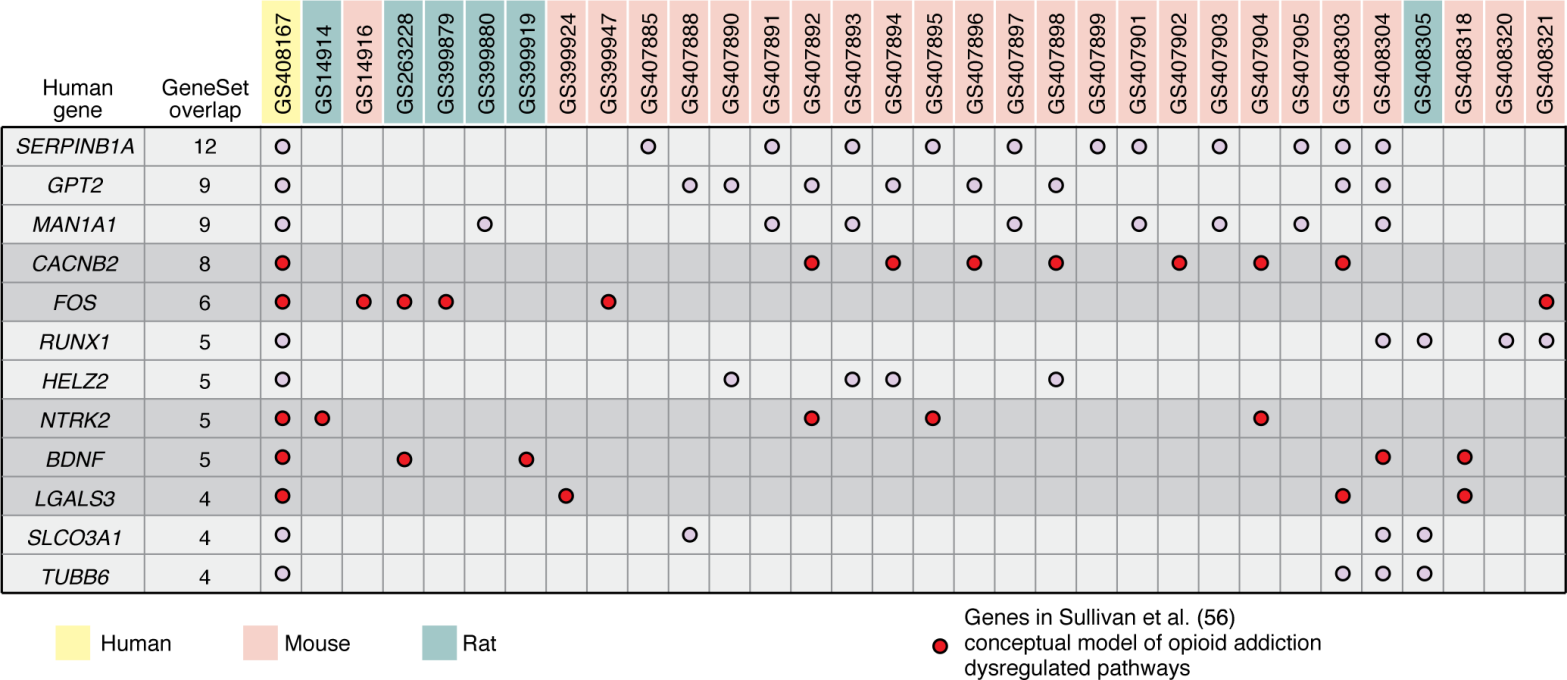
Cross-species analysis of independent datasets has the potential to nominate novel opioid addition–associated genes. In this example analysis, we identified overlap between a human opioid addiction–associated gene network (Sullivan et al., ref. 56 and 45 independent GeneSets collected from model organism experiments associated with opioid exposure (see Supplemental Table 1) within Geneweaver. We used a threshold requiring a gene nominated by Sullivan et al. (56) to be associated with an opioid exposure phenotype in at least three model organism GeneSet results. This analysis identified 12 such genes from among the 205 network genes examined (shown in column 1). (Of the 211 genes in the network identified in Sullivan et al., ref. 56, two were not present in Geneweaver and four did not have homologs in mice or rats, leaving 205 genes to be examined.) Note that candidate genes identified by this and related analyses would require functional validation in a controlled, repeatable environment.

Rodents share most of their coding genome with humans and present many of the same substance use behaviors (reviewed in refs. 61, 62), including those associated with OUD (63–66). Established diverse laboratory populations of mice (i.e., Diversity Outbred, Collaborative Cross, and recombinant inbred strains) and rats (i.e., Hybrid Rat Diversity Panel, Heterogeneous Stock Rats) include a wealth of genetic heterogeneity and carefully curated inbred lines that enable robust, repeatable manipulative studies. Importantly, these rodent models enable a multidimensional networked approach that integrates neural transcriptomics, including single-cell (67, 68) and spatial transcriptomics (69), to identify distinct cell-type-specific signaling pathways within well-annotated genomic backgrounds during various opioid exposure conditions. For example, mice exposed to interrupted bouts of morphine show greater changes in transcriptional pathways than those with continuous dosing (70). Moreover, sampling from six brain regions following acute and chronic heroin exposure, withdrawal, and relapse revealed distinct condition- and tissue-specific transcriptional changes affecting several biological domains in mice. For example, extracellular matrix (ECM) remodeling throughout the reward circuit is considered a driver of OUD in humans, and this work in mice identified heroin-induced enrichment of genes related to ECM, shedding new light on this process (71). Human brain samples are necessarily only acquired postmortem, yet comparing RNA-Seq data from opioid misusers who had died following overdose with those from individuals acting as controls who died of other causes identified transcriptional correlates between humans and mice; then, using published GWAS data, these findings were associated with genetic variants in human populations. Importantly, this analysis identified two genes, *FMO2* and *E2F1*, with regulatory changes in multiple brain regions that make them promising targets for treating OUD (71). Open-access resources, such as Geneweaver (72–74) and GeneNetwork (75), facilitate and democratize integrative systems genetics approaches across species to examine evidence linking variants and genes to outcomes across species. To illustrate the value of such cross-species lookup of results, we examined the overlap between an opioid addiction–associated gene network identified in a human multi-omic study (see above and Sullivan et al., ref. 56) with 45 independent GeneSets associated with opioid exposure collected from experiments with mice and rats that are included in Geneweaver (Supplemental Table 1; supplemental material available online with this article; https://doi.org/10.1172/jci.172886DS1). We used a threshold requiring a gene nominated in ref. 56 to be associated with an opioid exposure phenotype in at least three model organism GeneSet results. We identified 12 such genes from among the 205 network genes examined (Figure 2). Overlap of human multi-omic results with model organism results highlights the potential to generate hypotheses about human opioid addiction–associated genes that are functionally altered by opioid exposure under controlled experimental conditions using cross-species dataset analysis**.** However, functional validation of such hypotheses likely requires application of gene editing technology to model systems in a controlled, repeatable environment (reviewed in refs. 76, 77).

Emerging epigenetic and transcriptomic patterns associated with opioid overdose in humans have begun to elucidate the regulatory changes that occur in the brain with opioid use (reviewed above), yet the results from human studies can be complicated by the use of multiple drugs and variation in the interval from death to tissue preservation (25, 26, 78, 79). Use of multiple drugs is common among individuals with OUD; thus this is an important aspect of the underlying pathology of OUD, yet toxicology reports that document such polydrug use may not be available, particularly when studies use publicly available data (e.g., ref. 43). Work in diverse inbred mouse populations has demonstrated that opioid overdose is a heritable genetic trait (80). A recent study revealed that a cell-type-specific transcriptional response to oxycodone differed when a second drug was introduced to forebrain organoids (81), highlighting the need for studying opioids alone and in combination with other commonly used substances, which can be best executed in animal models. Model systems can add context to these studies by dissecting the effects of single and multiple drug exposure and the effects of postmortem interval. They can also allow sample collection to occur at key time points, such as during acquisition or relapse of opioid-seeking behavior. For example, work in rodents has shed new light on the molecular mechanisms underlying the ECM’s role in regulating the analgesic and rewarding effects of opioids, and across multiple stages of opioid addiction–like behavior (82), which helps to identify highly specific therapeutic targets that can be validated across a wide range of experimental paradigms in controlled environments in model systems.

## Discussion

To address the continuing opioid crisis and identify targets for new treatment development, a much deeper understanding of the biology underlying opioid addiction is needed. Through modern omics approaches, the field is making meaningful headway in identifying variants, genes, and pathways associated with opioid addiction phenotypes.

As the number of increasingly large GWAS of opioid addiction phenotypes has grown in the last few years, the number of genome-wide significant variants has increased. Initially limited to one or two variants, the largest meta-analysis identified 14 OUD-associated variants (17). Taken together, the individual GWAS results and various approaches to assessing the genetics shared between opioid addiction and other phenotypes, the current studies suggest that the strongest variant-level associations (e.g., those in *OPRM1* and *FURIN*) may be specific to opioid addiction, while weaker signals (e.g., in and around *RABEPK*) may be shared with other SUDs and psychiatric disorders. The most important steps to advance the identification of variants associated with opioid addiction are the most obvious: the field needs much larger and more ancestrally diverse GWAS cohorts and needs to conduct sex-stratified analyses, given differences in the epidemiology and risk profiles for opioid addiction by sex (19, 20), and analyses that extend to more phenotypes such as treatment response.

Across the studies of postmortem human brain, regulation of hundreds of genes appears to be associated with opioid addiction–relevant phenotypes, which have been identified primarily in studies of gene expression but also DNAm. However, the number of studies is limited. There is substantial heterogeneity of both phenotypes and reporting of results that limit comparisons, and sample sizes are small. Thus, it is unknown how robust many of these associations are. Despite these limitations, some gene dysregulation associated with opioid addiction was observed across omics data types (e.g., *DUSP* genes, *EGR* genes, *ARC,* *ARRDC3,* and *RERE*). Additionally, meta-analyses and a higher level of biological aggregation of genes into pathways have shown some consistency across studies, highlighting decreased expression of genes in the TrK/ERK/MAPK signaling pathways.

Examining the overlap of results sequentially across omics data types has important value for interpreting association findings and supporting the robustness of observed associations. However, this approach does not allow us to derive the biological connections between the identified biological features, particularly if the connections are not already established as known annotated biology. We can make these connections and substantially enhance the discovery of biological features associated with opioid addiction through concurrent analyses of multi-omic data that use systems biology approaches, such as those employed by Sullivan et al. (56), which linked GWAS findings (e.g., *OPRM1* and *FURIN*) with gene dysregulation findings (e.g., *DUSP* genes, *EGR* genes) based on an empirically derived dense gene network that highlights dysregulation of ERK/MAPK signaling, Akt, and BDNF in opioid addiction. Such workflows, which encompass network biology and structural biology integration to explore multi-omics data, can reveal intricate biological relationships and functions that are crucial to understanding the mechanisms underlying opioid misuse, addiction, and associated overdose deaths.

Omics studies in human populations are increasingly identifying biological features statistically associated with opioid addiction, a critical step in discovery. Bioinformatic evaluation of gene annotation is an important first step in suggesting biological interpretations of the role of identified biological features in opioid addiction. However, in human populations, phenotypic heterogeneity, the high prevalence of polysubstance use, and the broad psychiatric comorbidity with opioid addiction complicate the mechanistic interpretation of these statistical associations. Concurrent multi-omics studies further flesh out the complex connections among biological features associated with opioid addiction, resulting in more complete mechanistic hypotheses. Ultimately, though, experiments in model organisms need to test these omics-derived hypotheses to establish a mechanistic understanding of how they work. To date, most model organism studies of opioid addiction–related phenotypes have focused on hypotheses derived from other model organism studies rather than omics-based findings from human studies. Examination of cross-species overlap in genes associated with opioid addiction–relevant phenotypes in this Review (Figure 2) and in others (24) suggests that some findings in humans are supported by experimental results in model organism opioid exposure studies. However, model organism studies also suffer from a high degree of heterogeneity in phenotypes, study designs, and drugs tested, which limits how well such a gene overlap analysis can evaluate biological feature discoveries. Intentionally designing model organism experiments to test gene- and mechanism-based hypotheses derived from omics studies in humans is likely to yield much stronger tests of mechanisms that underlie the identified statistical associations. Furthermore, it is quite possible that cross-study and cross-species analyses focused on mechanistic overlap rather than specific gene overlaps will be more fruitful.

Finally, the advent of drug repurposing/compound-target pair databases (83–86) and tools (87) to leverage these data for prioritizing follow-up of gene lists from omics studies is an important development for the field. These resources will accelerate the translation of omics findings to the investigation of compounds for use in clinical care. Such analyses may become a standard follow-up to discovery analyses (10, 16–18, 29, 56, 59). A recent evaluation of potential repurposing of drugs to treat opioid addiction based on their targeting of genes associated with opioid addiction highlighted genes such as *OPRM1* and *DRD2* that are targeted by many drugs as well as some genes (e.g., *SST* and *BDNF*) that are known targets of single drugs (e.g., cysteamine and esketamine, respectively) (87). Ranking and expert review of such compound-target pairs may be another criterion by which to prioritize genes for mechanistic studies in model organisms.

## In summary

The field of substance use disorder omics has entered an exciting time where discovery of robust variant, gene, and pathway associations with opioid addiction are increasing rapidly. Among the set of genes associated with opioid addiction across omic studies that are well characterized in the catalog of gene annotation, some key receptors (e.g., GCPRs, GABA, and TrkB) and pathways have emerged, including RTK/ERK/MAPK signaling pathways, which suggest an important role of synaptic plasticity and neuronal signaling in opioid addiction (29, 56). Suggesting similar endpoints, multiple studies of gene dysregulation identify glial cell dysregulation associated with opioid addiction that affects neuronal plasticity (25, 26). The orexin pathway genes associated with opioid addiction also stand out as potentially important (29), given recent randomized clinical trials of medications for treating opioid addiction that target this pathway (e.g., suvorexant and lemborexant; NCT05546515, NCT04262193, NCT04287062, NCT05829655, NCT05145764, NCT04818086).

However, the subset of well-annotated genes is substantially smaller than the set of opioid addiction–associated genes; this larger set of genes falls into the so-called ignorome (88, 89). The lack of information on the biological function of ignorome genes biases researchers’ interpretation of their findings toward the biology that is known, reinforcing a focus on searching under well-illuminated lampposts. High-throughput means of evaluating the functional roles of the increasing number of opioid addiction–associated genes, particularly those in the ignorome, will be key to understanding the biological drivers of opioid addiction and prioritizing potential targets for drug development.

In addition to more expansive functional annotation of genes expressed in the human brain, larger sample sizes for all omics data types, expanded coverage of brain regions, and more refined biological signals (e.g., single nuclei omics, alternative gene splicing, and structural variant analyses) are all important factors that will accelerate progress. Continuing to leverage the agnostic discovery power of omics and placing it within the context of functional neurobiology will push us forward to much-needed, field-changing breakthroughs and the identification of actionable targets for drug development and new treatments for this devastating brain disease.

## Supplementary Material

Supplemental table 1

## Figures and Tables

**Table 2:**
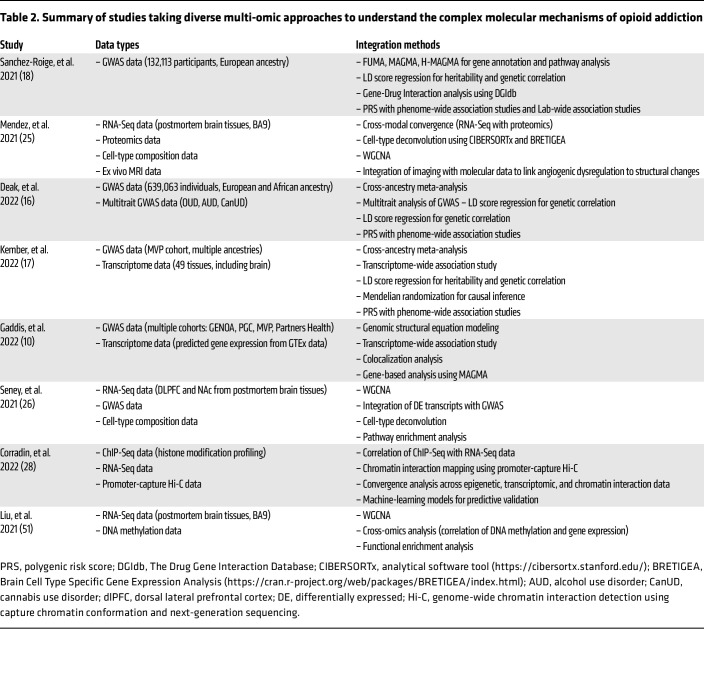
Summary of studies taking diverse multi-omic approaches to understand the complex molecular mechanisms of opioid addiction

**Table 1:**
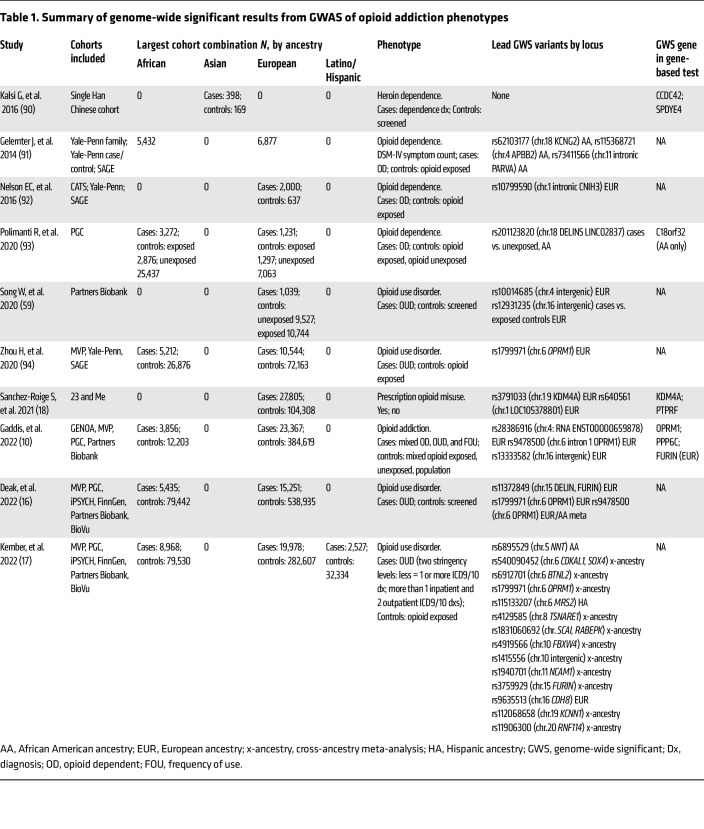
Summary of genome-wide significant results from GWAS of opioid addiction phenotypes

## Version 1 10/15/2024

Electronic publication
